# Supplementary material for: How to Address Consumers’ Concerns and Information Needs about Emerging Chemical and Microbial Contaminants in Drinking Water; The Case of GenX in The Netherlands
Source: Int J Environ Res Public Health. 2021 Oct 11;18(20):10615. doi: 10.3390/ijerph182010615 (PMC8535398; doi:10.3390/ijerph182010615)
Supplement: Supplementary file 1 [file ijerph-18-10615-s001.zip › ijerph-1368681-supplementary.pdf]

Supplementary Material to the Research Article

How to address consumers' concerns and information needs about emerging chemical and microbial contaminants in drinking water; the case of GenX in the Netherlands

Liesbeth Claassen[1], Julia Hartmann[1,2] and Susanne Wuijts[1,3]

[1] National Institute for Public Health and the Environment (RIVM), the Netherlands
[2] Delft University of Technology, the Netherlands
[3] Utrecht Centre for Water, Oceans and Sustainability Law, Utrecht University, the Netherlands
[*] Correspondence: julia.hartmann@rivm.nl

Texts and infographic used in the experiment

Three conditions were tested in the experiment:

- Condition 1: Existing website text
- Condition 2: Alternative website text
- Condition 3: Alternative website text and infographic.

Each condition started with basic information on Dutch drinking water. Below, we present the text both in Dutch and as a literal translation into English to avoid the introduction of language with a slightly different meaning. This could imply, however, that the use of the English language is somewhat 'rustic'.

Basic information

Dutch

Het drinkwater in Nederland is van goede kwaliteit. Wel is er reden tot zorg over de kwaliteit van de bronnen voor drinkwater; oppervlaktewater, grondwater of duinwater. In deze bronnen voor drinkwater kunnen resten van medicijnen, bestrijdingsmiddelen of chemische afvalstoffen van de industrie zitten. De drinkwaterbedrijven proberen bij de productie van drinkwater zoveel mogelijk van deze stoffen uit het water te halen door zuivering, maar met sommige stoffen lukt dat niet helemaal. Eén van die stoffen is GenX, dat gebruikt wordt om coatings voor bijvoorbeeld pannen te maken. Deze stof is in hele hoge doseringen mogelijk kankerverwekkend voor de mens.

English

The drinking water in the Netherlands is of high quality. There is however concern about the quality of the resources for drinking water production, such as surface water, groundwater and dune water, as traces of pharmaceuticals, pesticides and industrial chemicals have been identified in these drinking water resources. Drinking water companies try to remove these contaminants as much as possible during treatment, but some contaminants cannot be completely removed. One of these contaminants is GenX. This contaminant is used, for example, in non-stick coatings in frying pans. In high dosages, GenX is possibly carcinogenic to humans.

Existing website text

Dutch

[Published on https://www.rivm.nl/genx/drinkwater]

Uit onderzoek van het RIVM (Rijksinstituut voor Volksgezondheid en Milieu) blijkt dat GenX in drinkwater bij drie Nederlandse drinkwaterbedrijven verhoogd is, maar veilig. De

concentratie GenX in drinkwater zal in de toekomst de richtwaarde nét niet overschrijden bij de hoeveelheid GenX die nu door bedrijven, conform de vergunning, wordt uitgestoten. Het RIVM heeft deze richtwaarde afgeleid op basis van de dagelijkse toelaatbare inname van de GenX stoffen bij levenslange inname via drinkwater.

Indicatieve richtwaarde

Het RIVM heeft in 2016 een indicatieve richtwaarde afgeleid voor GenX in drinkwater. Deze waarde ligt op 150 nanogram per liter water. Bij het bepalen van deze waarde heeft het RIVM gekeken naar de dagelijkse toelaatbare inname van de GenX stoffen bij levenslange inname via drinkwater. Er wordt hierbij ook rekening gehouden met inname via andere routes, zoals lucht. Bij een concentratie van 150 nanogram per liter drinkwater zijn er geen negatieve gevolgen voor de gezondheid te verwachten.

English

Research by the Dutch National Institute for Public Health and the Environment (RIVM) shows that levels of GenX in drinking water from three Dutch drinking water companies are elevated, but safe. In the future, the concentration of GenX in drinking water is expected to, only barely, not exceed the guideline value for the amount of GenX that is currently emitted by industries, in accordance with the permit. RIVM based the guideline value on the acceptable daily intake of GenX over a lifetime intake of drinking water.

Indicative guideline value

RIVM defined an indicative guideline value for GenX in drinking water in 2016. This value was 150 nanogram per litre of water. RIVM based the health-based guideline value on the acceptable daily intake of GenX over a lifetime intake of contaminated drinking water. Exposure to GenX via other exposure routes such as air was also taken into account. At a

concentration of 150 nanograms per litre drinking water, no negative health effects are expected.

Alternative website text

Dutch

Het drinkwater van drie drinkwaterbedrijven in Zuid-Holland en Zeeland (Oasen, Evides en Dunea) bevat kleine hoeveelheden GenX. Deze stof is afkomstig van bedrijven die een vergunning hebben voor een beperkte uitstoot. De hoogst gemeten hoeveelheid GenX in het drinkwater is op dit moment 40 nanogram per liter (= 0,00000004 gram per liter). Dat is minder dan de veilige norm van 150 nanogram per liter die op basis van RIVM onderzoek is vastgesteld. Dit betekent dat ook bij levenslang dagelijks water drinken van deze drie bedrijven geen negatieve gevolgen voor de gezondheid door GenX te verwachten zijn.

English

The drinking water of three drinking water companies in the provinces Zuid-Holland and Zeeland (Oasen, Evides and Dunea) contains small quantities of GenX. This contaminant originates from industrial companies who have a permit for limited emission. At this moment, the highest concentration of GenX measured in drinking water is 40 nanograms per litre (= 0. 00000004 gram per litre). This is below the safe guideline value, which is based on RIVM research, of 150 nanograms per litre. This means that even over a lifetime intake of drinking water from the three mentioned drinking water companies, no negative health effects due to GenX are expected.

Infographic

Dutch

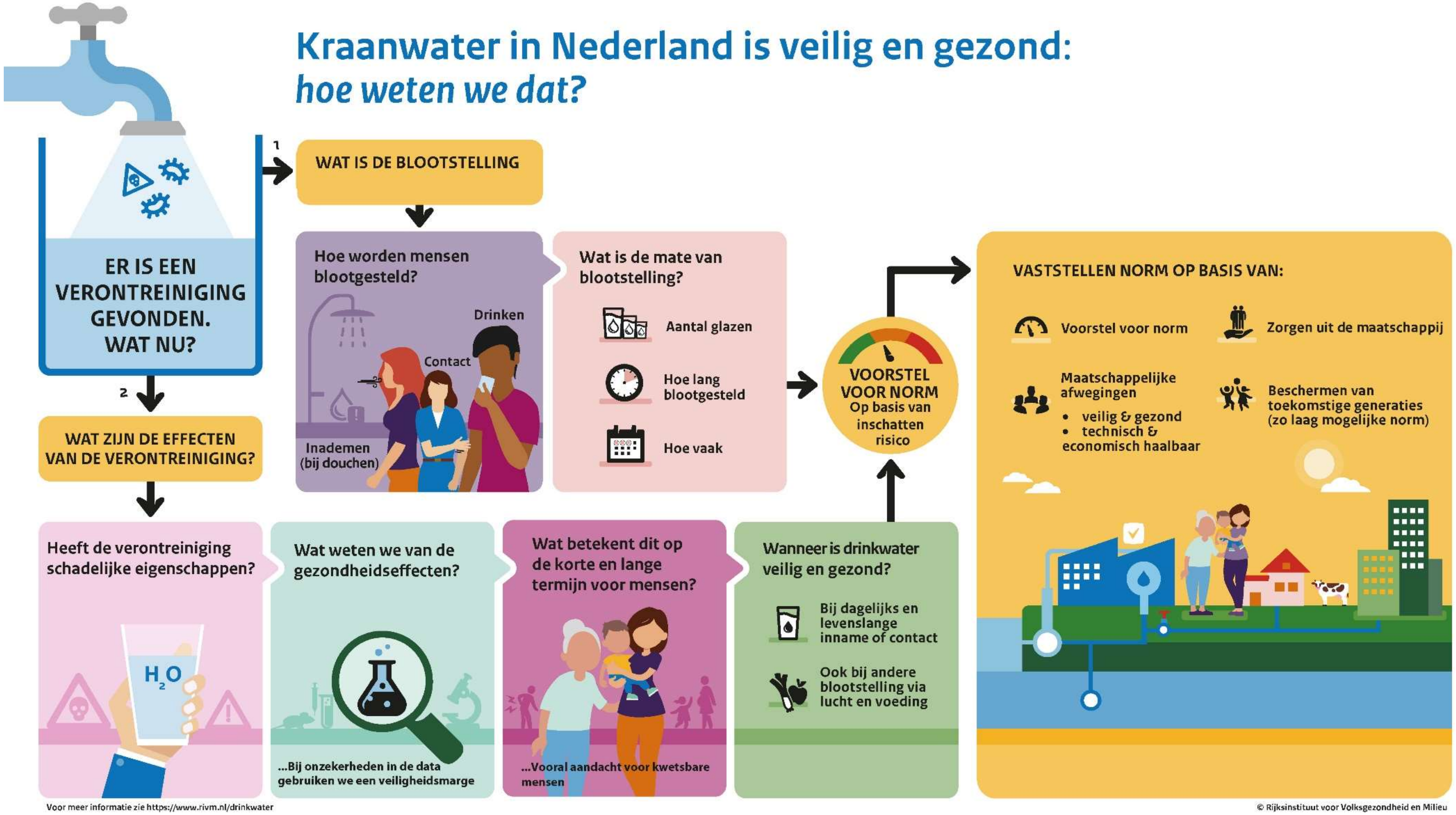

English

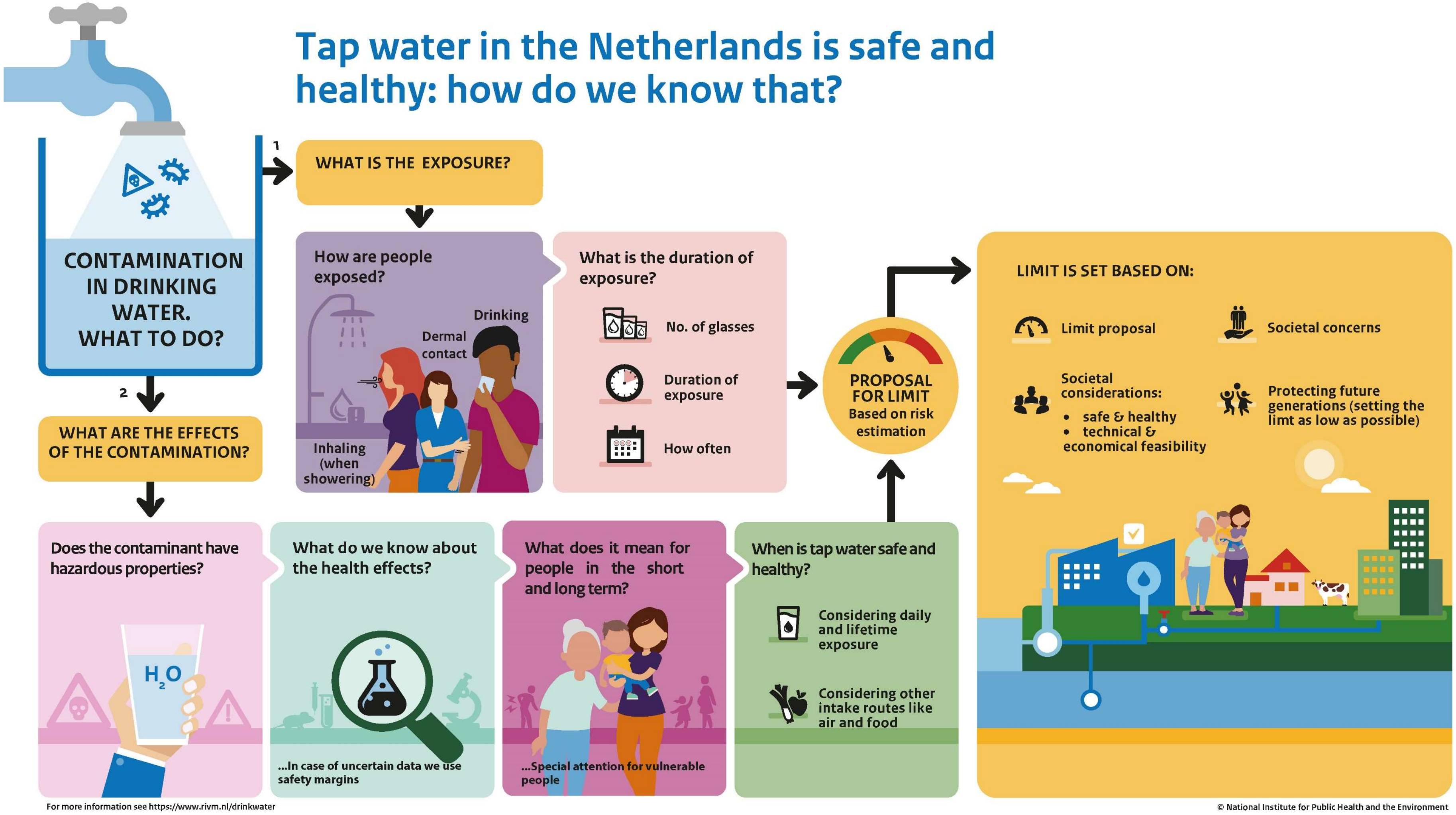
Tap water in the Netherlands is safe and healthy: how do we know that?
CONTAMINATION IN DRINKING WATER. WHAT TO DO?
1
WHAT IS THE EXPOSURE?
How are people exposed?
Drinking
Dermal contact
Inhaling (when showering)
What is the duration of exposure?
No. of glasses
Duration of exposure
How often
PROPOSAL FOR LIMIT
Based on risk estimation
LIMIT IS SET BASED ON:
Limit proposal
Societal concerns
Societal considerations:
• safe & healthy
• technical & economical feasibility
Protecting future generations (setting the limt as low as possible)
2
WHAT ARE THE EFFECTS OF THE CONTAMINATION?
Does the contaminant have hazardous properties?
H2O
What do we know about the health effects?
...In case of uncertain data we use safety margins
What does it mean for people in the short and long term?
...Special attention for vulnerable people
When is tap water safe and healthy?
Considering daily and lifetime exposure
Considering other intake routes like air and food
For more information see https://www.rivm.nl/drinkwater
© National Institute for Public Health and the Environment
